# The Growth and Scope of Voluntary Hospitals in London

**Published:** 1897-11-27

**Authors:** 


### THE GROWTH AND SCOPE OF VOLUNTARY HOSPITALS IN LONDON.

(From a Correspondent.)

### XVI.—ANÆSTHETICS.

Hospital development during this reign is marked by two white stones recording successive achievements, which together have effected an entire revolution in hospital work and life. It is the great glory of the age that it has forged weapons mighty to subdue not only pain, but even death itself, those two haunting terrors of the wards which in bygone days it often seemed to the pitiful were but invoked on the incoming patient by all the wealth and skill expended to relieve his sufferings. The gulf which separates the operations of the first years of the reign from the operations performed in the present day is immeasurable, but the change of feeling in surgeon and patient alike marked by the 1849 era of anæsthetics is hardly greater than the difference in mortality involved in the antiseptic methods introduced a score of years later.

The part played by the hospitals in both these great epochs is well worth studying. It is well known that Sir Humphry Davy, as early as 1800, experimented with nitrous oxide gas, and suggested its use in surgical operations. The suggestion, however, bore no fruit, and the honour of the first systematic application of anæsthetics to surgery must be assigned to America. On December 11th, 1844 (the dates are jealously quoted from one side and another), a certain Dr. Wells, of Hartford, U.S., extracted a tooth from a patient under nitrous oxide gas. He published an account of the proceeding, which attracted a good deal of attention, and an enormous audience gathered to see a second operation of the same kind which he undertook to perform in Boston. For some reason or other, the action of the gas failed, and Dr. Wells was hooted out of the town; his experiments were derided, and he was completely discouraged from further attempts.

Two years later, on September 20th, 1846, Dr. Morton, a former pupil of Dr. Wells, after many experiments with sulphuric ether, extracted a tooth from a patient under the influence of this anæsthetic. The discovery was hailed with acclaim, and before the end of the year successive operations were performed under sulphuric ether with unvarying success at the General Hospital, Massachusetts.

Early in December of that year the news of the anæsthetic had reached the ears of Liston. It was believed at first that the action of sulphuric ether was so transitory as to render it of use only in operations which could be concluded in a few moments, and Liston, as the most rapid operator of his own or any age, conceived that it might be of special service to him. On December 21st, 1846, he made his first trial, and selected two specially trying operations at University College Hospital, where he held the appointment of consulting surgeon, for the administration of the vapour of ether. The result in both cases succeeded beyond expectation, and the ecstacy of the newly awakened patients on hearing that the dreaded operation was actually concluded is briefly indicated in the letter from Liston relating the details of the whole experiment, which appeared in the *Lancet* for January 2nd, 1847. That was a momentous number of the journal, containing, as it did, in addition, a paper by Dr. Bigelow, of Massachusetts, with a detailed account of the new discovery and a description of the right method of applying it for the relief of suffering. University College Hospital thus carries off the honour of authorising the first operation in England under anæsthetics. Very speedily indeed one hospital after another followed suit, and the weekly record of "operations without pain," at first minutely detailed and eagerly studied by half-incredulous readers, became a regular feature until their number grew too great for insertion; and finally, more quickly than could have been supposed possible, anæsthetics settled into a commonplace, and an occasional failure alone was worthy of remark.

King's College Hospital was the next after University to try anæsthetics. Fergusson, the consulting surgeon, contributes a record of nine cases, from December 31st, 1846, to January 9th, 1847, in which operations were performed under vapour of ether. At St. Thomas's, on January 9th, and at Westminster, on January 11th, several patients were successfully operated on under the new anæsthetic, one of the cases at St. Thomas's being a child. At Guy's, on January 12th, Mr. Morgan operated with the aid of the vapour, the theatre being densely crowded with students and professional men. At St. George's, on January 14th, an amputation was attempted and successfully carried through, and on the same day the first experiment was made at Charing Cross Hospital. At St. Bartholomew's, on January 16th, three cases were placed under vapour of ether, but one was a complete failure, owing, it was thought, to previous habits of intemperance. Again we read that the operating theatre was much crowded by practitioners as well as students, and it is perfectly clear that men in private practice flocked to the hospitals to gain the knowledge, nowhere else attainable, of the inestimable boon they were eager to introduce among patients in their own homes.

Throughout the spring and summer the use of vapour of ether as an anæsthetic spread throughout the country. An impudent attempt was early made by an American to claim a patent for its administration, and force every surgeon desirous of using it to buy the privilege. It is curious to read the grave arguments of learned counsel against the possibility of patenting the administration of a given quantity of any particular drug, which was, in fact, the American's pretension. But this particular anæsthetic had not come to stay. Its imperfections became very apparent as its use was extended. The strong and offensive odour which clung to the persons of those who employed it was a minor drawback, involving, nevertheless, considerable discomfort. But its frequent failure under some conditions to produce entire unconsciousness made it a very uncertain instrument, and it is doubtful if it could ever have come into universal use with the appliances which were then available.

Meantime, through the autumn months of this memorable year, James Young Simpson, Professor of Midwifery at Edinburgh, whose dream it had been since childhood to lessen the sufferings of patients under the surgeon's knife, had been experimenting with a new medium. On November 4th, 1847, he made the first deliberate trial of inhaling chloroform, and speedily satisfied himself and others of the importance of the new discovery. The earliest operations under chloroform were performed in the Royal Infirmary, Edinburgh, and a letter from Simpson, of November 20th, to the *Lancet* described the new anæsthetic, and urged its adoption in surgical practice. But the discovery had already reached London. At St. George's Hospital Dr. Snow operated on a patient under chloroform on November 18th, and two days later operations were performed at both Westminster Hospital and St. Bartholomew's under the same conditions. The discovery attained widespread publicity almost instantaneously; but, although it may be said to have revolutionised surgical practice, no such sensation attended its introduction as ensued upon the earlier operations in the year under the first practical anæsthetic. Starting in hospital practice, where within a few days almost it gained an established place, it spread to private practice, and mainly through the instrumentality of its discoverer soon became a new and most potent agent in midwifery. It was principally in the latter branch of practice that its use was denounced from the pulpit and elsewhere as dangerous to health, morality, and religion.

In making known these important discoveries, in vying one with another in the ready adoption of ideas so startling and methods so novel, the voluntary hospitals performed a work of inestimable benefit to the community. The freedom of action permitted by their constitution to the principal medical officers was and is a most important factor in promoting research, while the keen emulation resulting from the entire independence of the various kindred institutions lent a spur to progress which no State-regulated system could ever supply.

